# Relationship of salivary CD14 concentration with dental caries in young children

**DOI:** 10.4317/jced.53796

**Published:** 2017-08-01

**Authors:** Saurabh Kumar, Shobha Tandon, Rashmi Nayak, Runki Saran, Kalyana-Chakravarthy Pentapati

**Affiliations:** 1 Department of Pedodontics and Preventive Dentistry, Manipal College of Dental Sciences, Manipal University, Manipal; 2 Centre of Studies for Paediatric Dentistry and Orthodontics, Faculty of Dentistry, University Technology MARA, Malaysia; 3 Faculty of Dentistry, Melaka Manipal medical college, Manipal University, Manipal; 4 Department of Public Health Dentistry, Manipal College of Dental Sciences, Manipal University, Manipal

## Abstract

**Background:**

Dental caries is a chronic disease among children and there is need for studies assessing the caries risk factors using saliva. This study aimed to evaluate the association of salivary soluble CD14 and dental caries in young children.

**Material and Methods:**

A cross-sectional study was done among 300, 3-6 year old school children of Udupi district. A total of 40 children who were caries free, with no past systemic illness or craniofacial anomalies and 40 children with dental caries with no history of dental treatment for caries, with no past systemic illness or craniofacial anomalies were included in control and test groups respectively. Salivary CD14 was evaluated using ELISA test.

**Results:**

The mean salivary soluble CD14 concentration was significantly higher in caries free (1.34±0.35 µg/ml) children than caries experienced (0.54±0.36 µg/ml) (*p*<0.001). There was significant strong negative correlation between number of decayed teeth and soluble salivary CD14 (r = -0.868, *P*< 0.001) among all the children. Similarly, sub-group analysis of caries experienced children also showed significant strong negative correlation between number of decayed teeth and soluble salivary CD14 (r = -0.774, *P*<0.001).

**Conclusions:**

Results obtained in our study suggested that salivary CD14 can be a indicator of dental caries in young children.

** Key words:**Caries, CD14, Children, Saliva.

## Introduction

Dental caries is the most prevalent dental disease affecting mankind. A remarkable reduction in the prevalence and severity of caries has been observed in many countries over recent decades. Fluoridation of public water supplies and wide use of dentifrices remarkably improved oral hygiene. However, dental caries remains a significant public health problem. A recent systematic review reported an increasing prevalence in developing countries (1).

The etiology and pathogenesis of dental caries are known to be multifactorial in which bacteria play an essential role. Among the large number of bacterial species harbored by the dental plaque, *Streptococcus mutans, Streptococcus sorbinus, Lactobacilli* and *Actinomyces* have been positively associated with dental caries (2). Saliva acts as the major host defense system and also maintains the balance between demineralization and remineralization.

Salivary proteins have been reported to have a role on the initiation of caries (3). Understanding the role of salivary proteins in caries development is a daunting task. Many of these proteins have multiple functions or share common functions or even sometimes have opposite functions (4,5). These proteins and glycoproteins present in saliva protect oral tissues, but knowledge about the role of human saliva in innate immunity is scant.

The innate immune system invades microorganisms through the recognition of pathogen-associated molecules. Salivary (sCD14) is a glycoprotein expressed predominantly on the surface of monocytes, macrophages and neutrophils. It plays a crucial role in the recognition of several microbial products, such as lipopolysaccharides (LPS), endotoxins and peptidoglycans, which are major components of cell wall of gram-negative and gram-positive bacteria (6,7). Previous studies have showed the expression of this factor in the saliva and some studies have showed significant association with dental caries experience (8) while some studies haven’t shown such an association (6). In view of this, we aimed to evaluate the association between salivary CD14 and dental caries experience.

## Material and Methods

This comparative cross-sectional study was done among school children of Udupi district. The study was approved by institutional ethics committee, Manipal University, India. Written informed consent was obtained from all the parents prior to the study.

A total of 300 children aged between 3 to 6 years were screened. A clinical examination of all the subjects participating in the study was carried out using a mouth mirror and probe by a single examiner. A sample size of 29 children were required which was based on the calculation with 90% power and an alpha of 0.05 for an effect size of 0.8. Sample size calculation was done based on the findings of Biria *et al.* (9). However, we have recruited a sample of 40 children in each group to have wider representation of caries teeth to evaluate the correlation with salivary CD14. Caries was examined by a single trained and calibrated examiner according to World Health Organization (1997) diagnostic criteria (10). Intra-examiner reliability as assessed by intra class correlation coefficient for decayed teeth was 0.98. A total of 40 children who were caries free, with no past systemic illness or craniofacial anomalies were included in control group. For study group, 40 children with dental caries with no history of dental treatment for caries, with no past systemic illness or craniofacial anomalies were included.

Children were instructed to refrain from eating, drinking, chewing candy or performing any means of oral hygiene at least one hour prior to collection of saliva. Children were asked to rinse their mouth with water. The collection of unstimulated saliva was done in a quiet room in the morning (11). Saliva was collected by passive drooling method into sterilized containers where child was seated with head slightly down and was asked not to swallow or move tongue or lips. The samples were transported on ice to the laboratory immediately for processing. Each sample was clarified by centrifuging at 4000g at 4°C for 10 mins and stored.

The sCD14 levels of the saliva were measured on thawed samples by ELISA method using sCD14 kit (Human sCD14 Quantikine kit, R and D system, Minneapolis, MN). The optical density was read at 450 nm and the sCD14 concentration (µg/ml) was determined according to the manufacturer’s instructions. After the collection of saliva, comprehensive treatment was provided for both the groups. The estimation of sCD14 by ELISA was performed by trained technician.

-Statistical analysis:

All the data was entered into SPSS version 15.0 software package (SPSS Inc, Ill, Chicago, USA). A *p*-value of <0.05 was consi-dered statistically significant. Comparison of mean soluble salivary CD14 concentration was done using Mann-Whitney U test. Correlation of decayed teeth with CD14 concentration was done using Spearman ranked correlation.

## Results

The mean age of the caries free and caries experienced children were 4.75±0.84 and 4.8±0.79 years respectively. A total of 21 males and 19 females participated in each group. The mean dmft of the caries experienced children was 5.05±2.66 (Range=1-11). The mean dt and mt among the caries experienced children were 4.88±2.57 and 0.22±0.58 respectively.

**Figure 1:**
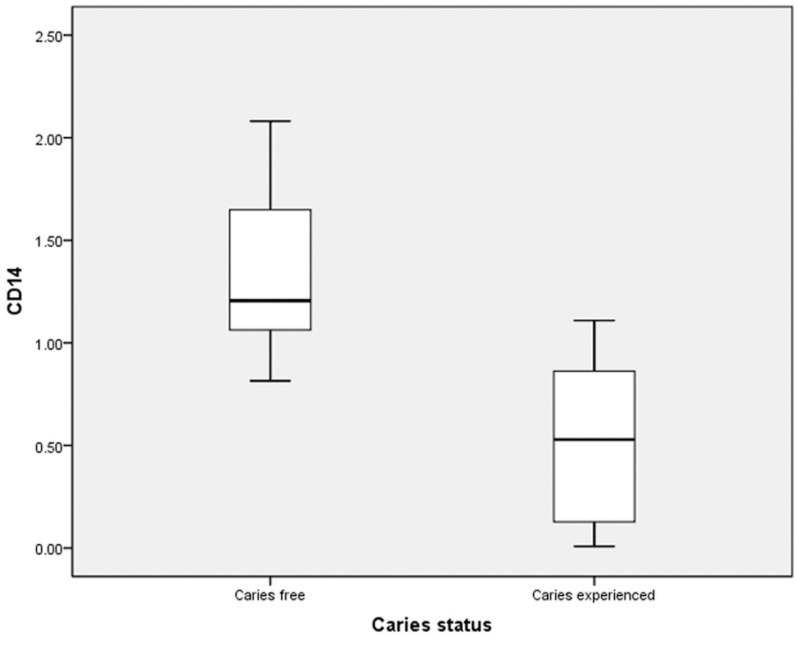
Mean salivary CD14 concentration between caries free and experienced children.

The mean salivary soluble CD14 concentration was significantly higher in caries free (1.34±0.35 µg/ml) children than caries experienced (0.54±0.36 µg/ml) (*p*<0.001) ( Table 1, Fig. 1). There was significant strong negative correlation between number of dmft and soluble salivary CD14 (r = -0.868, *P*<0.001) among all the children. Similarly, sub-group analysis of caries experienced children also showed significant strong negative correlation between number of dmft and soluble salivary CD14 (r = -0.774, *P*<0.001).

**Table 1:**
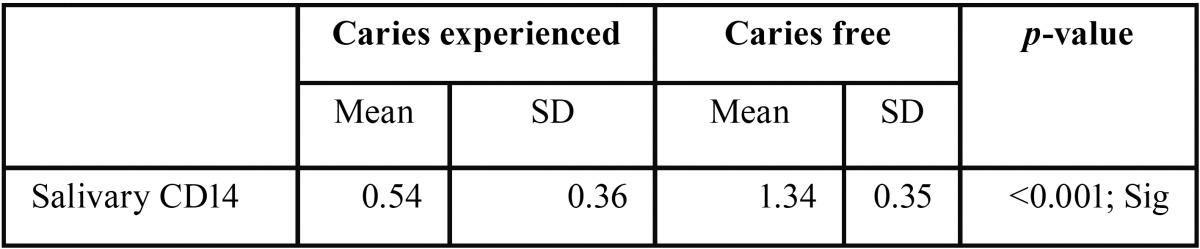
Comparison of mean salivary CD14 concentration between caries free and experienced children.

## Discussion

The expression of CD14 by human salivary glands in a functionally active form has been reported previously. Bas *et al.*, suggested that soluble CD14 is a glycoprotein which functions in Lipopolysacharide/cell-wall products signaling, by controlling the immune system level of response (12). ELISA can be used to analyze the soluble CD14 concentration in saliva.

Although the function of soluble salivary CD14 in human disease has not yet been clear, a potential pathogenic role of soluble CD14 in several infectious diseases has been proposed. Elevated levels of soluble CD14 have been found in non-infectious and infectious diseases such as polytraumatised and severely burned patients (13), rheumatoid arthritis (14). systemic lupus erythematosus (15), septic shock (16), tuberculosis with or without HIV infection (17).

In our study, an inverse relationship between dental caries and soluble salivary CD14 concentration was observed which was in accordance with previous study (6) which used western blot method. In present study, we analyzed soluble CD14 quantitatively using ELISA test as it is more sensitive test than western blot test. Similar method was used in previous studies but have reported contradictory results (8,9). Biria *et al.* (9) reported that the difference in the result could be due to the variation in the age groups since specific immune responses have not yet been developed completely in 3-5 year old children, the increase in sCD14 level in saliva may be a reparative response of immune system to the inadequacy of such immunoglobulins as IgA and IgG. Few researchers (6,12) have reported that sCD14 can regulate humoral and cellular immune responses through interaction with B and T cells and have a role in natural immunity and it was identified to have a preventive function against periodontal diseases. From the findings of our study, it can be concluded that higher concentration of sCD14 might have prevented the individual from caries development (18).

The difference in levels of soluble CD14 in caries experienced and caries free groups indicates the role of innate immunity in oral cavity. Pugin *et al.* (19) proposed that soluble CD14 plays a crucial role in the initiation of immune responses by recognition of several microbial products, such as lipopolysaccharide (LPS), endotoxins and peptidoglycan, which are major components of gram-negative and gram-positive bacteria, respectively. Also, Sugawara *et al.*, (20) found in their study that saliva contains abundant bio-active CD14 from salivary glands in a soluble form. This suggests that soluble CD14 is important for the maintenance of oral health. Based on the results of the study, it can be concluded that salivary CD14 can be used as indicator of dental caries. In view of inconsistent findings on the potential role of sCD14 and its relationship with caries further research is recommended.
